# Outcome of photodynamic therapy with Rose Bengal in conjunction with topical PHMB and chlorhexidine combination in Acanthamoeba keratitis

**DOI:** 10.1186/s12348-025-00466-w

**Published:** 2025-03-05

**Authors:** Bhupesh Bagga, Lakshminarayanan Gowtham, Lalit Kishore Ahirwar, Debkuntal Sen, Saumya Jakati, Md Hasnat Ali, Savitri Sharma

**Affiliations:** 1 The Ramoji Foundation Centre for Ocular infection, Shantilal Shanghvi Cornea Institute, Hyderabad, India; 2 Dr. Chigurupati Nageshwara Rao Ocular Pharmacology Research Centre, LV Prasad Eye Institute, Hyderabad, India; 3 Jhaveri Microbiology Centre, Hyderabad, India; 4 Clinical research fellow, LV Prasad Eye Institute, Hyderabad, India; 5 Ophthalmic Pathology Laboratory, Kallam Anji Reddy Campus, LV Prasad Eye Institute, Hyderabad, India; 6 Department of Computational Bio-Statistics and Data Sciences, LV Prasad Eye Institute, Hyderabad, India

**Keywords:** *Acanthamoeba* Keratitis, Microbial keratitis, Photo-dynamic antimicrobial therapy, Rose Bengal

## Abstract

**Purpose:**

To report the outcome of *Acanthamoeba* keratitis, with early addition of Photo-dynamic antimicrobial therapy with Rose Bengal (PDAT-RB) to the medical treatment (combination of 0.02% Polyhexamethylene Biguanide (PH)and 0.02% chlorhexidine(CH)).

**Methods:**

Patients were recruited based on the infiltrate size being < 8 mm and limited to the mid stroma, < 300µ, and confirmed microbiological diagnosis. Along with the continuation of PHMB + CH, patients were also treated with PDAT-RB twice with a gap of one week using 0.1% w/v RB and green LED (525 nm) array immediately after the confirmation of diagnosis.

**Results:**

A total of 14 patients were enrolled. All the enrolled patients received adjuvant PDAT-RB within 5 (2.5 to 11) days of diagnosis. The average diameter and median depth of the infiltrate were 5.7 ± 1.56(V), 5.9 ± 1.38(H) mm, and 250 (250 to 300)µ, respectively. The mean LogMAR visual acuity at the time of presentation was 2.52 ± 0.95. Out of 14 enrolled patients, infection was resolved in 12 (85.7%) patients, whereas 2 (14.3%) patients needed TPK. The median days to resolve were 110 (67 to 150) days. The final mean LogMAR Visual acuity at the end of the follow-up was 1.60 ± 1.3.

**Conclusion:**

The study demonstrates the effective resolution of *Acanthamoeba* keratitis when treated with early adjuvant photodynamic antimicrobial therapy using Rose Bengal (PDAT-RB).

## Introduction

*Acanthamoeba* keratitis [1–3] is one of the most challenging forms of microbial keratitis. The patients diagnosed at an early stage [4, 5] achieve faster and complete resolution with a combination of topical biguanides versus those of advanced stage [6]. However, because most of these cases closely resemble Herpes Simplex keratitis clinically and thus receive topical corticosteroids as a treatment and present in the delayed presentation to the clinic. These advanced cases, even with the combination of biguanides, worsen more than 50% [6, 7] of the time and require either urgent therapeutic keratoplasty (TPK) or Deep Anterior Lamellar Keratoplasty (DALK) [8] to control the infection. The poor outcome can be due to the advanced stage of keratitis at the time of presentation or poor response [3, 11] to medical treatment due to deeper involvement or lack of compliance due to the long duration of therapy, the use of corticosteroids or associated toxicity [12] of medications. Many of those who achieve clinical resolution cannot achieve good visual acuity and need further optical keratoplasty (OPK) [9, 10] for vision restoration. Other treatment options like [11, 13–16] voriconazole and miltefosine have been tried, with variable success and also associated with systemic side effects. Because of these issues, there is always a search for newer, more effective, and safer medical and alternative or adjuvant treatment needed for better management of *Acanthamoeba* keratitis with the goal of not only eradicating infection but also decreasing treatment duration and increasing final visual acuity to avoid urgent TPK. The use of Photoactivated chromatophore for keratitis-collagen cross-linking (PACK-CXL) [17] and Photodynamic therapy with photosensitizers [18] have been attempted in recent years in other forms of microbial keratitis with various successes. *Acanthamoeba* keratitis has been treated similarly in different in vitro and in vivo [20] models, substantially reducing infection.

In a case series by Naranjo et al. [18], this photodynamic antimicrobial therapy using Rose Bengal (PDAT-RB) decreased the need for TPK in 72% of cases using PDAT-RB. Its efficacy has also been observed in the animal model [20] of *Acanthamoeba* keratitis and has been shown to reduce the infection load with subsequent resolution. In the present study, we have introduced PDAT-RB in the early phase of the disease as it will aid in the early eradication of infection with subsequent faster recovery, better visual outcomes, lesser need for TPK, and improved patient compliance.

## Methods

The study was approved by the Institutional Review Board of LVPEI(LEC-BHR-P-10-20-535) and adhered to the Declaration of Helsinki. The study was also registered with the Clinical trials registry India CTRI (CTRI/2020/12/029504). Selection criteria included the overall diameter of the corneal infiltrate (ring, confluent or patchy) being equal to or less than 8 mm as measured by a slit lamp (Haag Streit slit lamp, USA) with the depth of the infiltrate, assessed with anterior segment Optical Coherence Tomography(OCT) (Optovue, Inc, Fremont, CA) to have less than 300µ or clinical evaluation of the depth with slit lamp. The exclusion criteria were (a) patients with posterior stromal or limbal involvement (either clinical or OCT-based), (b) with only one functional eye (other eye vision if less than 6/60 due to unmodifiable causes), (c) allergic to Rose Bengal, as tested by keeping the Rose Bengal solution in the conjunctival sac and observe the reaction (d) with any associated collagen vascular disease, (e) with a possible history of herpes simplex virus keratitis (f) with mixed infection. As this was a pilot trial, we did not calculate the sample size.

The diagnosis of *Acanthamoeba* keratitis was based on the microscopic observation of double-walled cysts of *Acanthamoeba* in corneal scrapings on potassium hydroxide with calcofluor white (KOH + CFW) mount and Gram stain, which was confirmed by growth of the *Acanthamoeba* in culture (Non-nutrient Agar enriched with *E. coli*). We also did a confocal microscopy on all patients to confirm the diagnosis at the time of presentation and follow-up.

Following the microbiological confirmation, all the patients were treated with a combination of hourly topical 0.02% w/v Polyhexamethylene biguanide (PH) and 0.02% w/v Chlorhexidine (CH). PDAT-RB was performed in all patients as soon as the microbiological diagnosis was confirmed. 0.1%w/v Rose Bengal (Cat. no-33000, Sigma-Aldrich, USA) was prepared in a balanced salt solution as per the standard procedure. Epithelial debridement was done 2 mm around the corneal infiltrate to help increase Rose Bengal absorption and remove debris potentially. Rose Bengal concentration on the eye was maintained with a soaked sponge, and the rose Bengal solution was continued every 2 minutes for 30 min. Irradiation was performed using a computer-controlled light source comprising a green LED array at 525 nm wavelength with a fluence of 5.4 Joules/cm^2^ in a dark room for 15 min. It was followed up every alternate day for examination. A similar procedure was repeated twice in all patients with an interval of 1 week period. The above protocol of PDAT-RB is according to the previously used at Bascom Palmer Eye Institute [18]. The light source used was identical and was supplied by the Ophthalmic Biophysics Center at Bascom Palmer Eye Institute, USA. The patients with evidence of clinical worsening were subjected to TPK, and the corneal button was sent for microbiological and histopathological examination. The criteria of clinical worsening were defined as an increase in the size of the infiltrate, extension into the deeper stroma, and an increase in the size of the hypopyon.

The primary outcome measure was the proportion of cases resolved on medical treatment (medical cure) [19], and the secondary outcome measure was the number of cases that underwent penetrating keratoplasty. Success was defined as complete clinical resolution of infection, defined as no evidence of epithelial staining, stromal cellularity, or infiltrate and quiet anterior chamber without anti- Acanthamoeba medications for more than one month, while failure was defined as worsening of infection with the need for surgical intervention. Both PHMB and Chlorhexidine were administered topically every hour for 3 weeks, followed by 2 h until complete clinical resolution. After the complete clinical resolution, the patient was followed up every two weeks, and the medications were reduced to 4 h for six weeks and then discontinued. Topical corticosteroids (1% w/v Prednisolone acetate) were also added as per the practice after a minimum of 2 weeks starting treatment with careful observation of clinical signs suggestive of response or worsening.

## Results 

Fourteen patients out of a total of 65 patients (65 eyes) seen from August 2020 to Sept 2022 with confirmed *Acanthamoeba* keratitis were enrolled based on the pre-defined inclusion criteria. Their demographical, clinical features and treatment profile are described in Table 1. Seven (50%) out of fourteen cases had a history of trauma while working in the agriculture field. At the time of presentation to the clinic, patients were using topical antifungals (*N* = 5), antibiotics(*N* = 8), antivirals (*N* = 1), anti-*Acanthamoeba*(*N* = 1), and corticosteroids (*N* = 7).

All the patients’ mean LogMAR visual acuity was 2.52 ± 0.95 at the presentation time. The median duration between the onset of symptoms from the time of presentation to the clinic was 30 (IQR, 20.5–30.5) days. The most common clinical sign was the presence of ring infiltrate in 10/14 (71.4%). The mean diameter of the infiltrate (in either form, that is, ring, diffuse cellularity, or multiple lesions) in vertical and horizontal meridian (from edge to edge) was 5.7 ± 1.56and 5.9 ± 1.38 mm, respectively. The median depth of infiltrate was 250 (250–300µm).

**Fig. 1:**
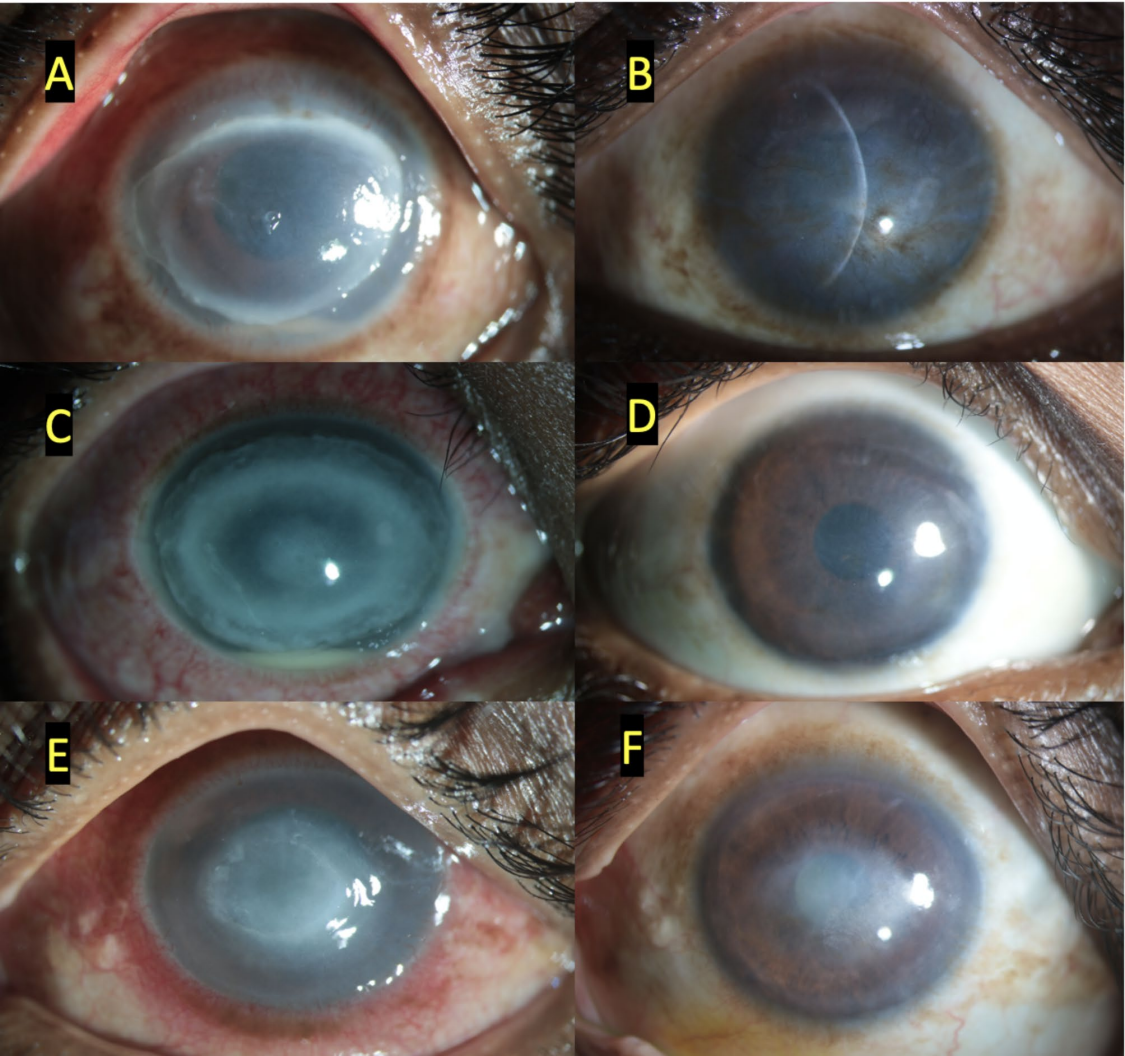
Shows 3 clinical pictures of three cases at the time of presentation (**A, C, E**) and at their last visit with complete clinical resolution (**B, D, F**). With the treatment of photodynamic antimicrobial therapy using Rose Bengal along with topical 0.02% w/v of Polyhexamethylene biguanide and 0.02% w/v of Chlorhexidine

**Fig. 2:**
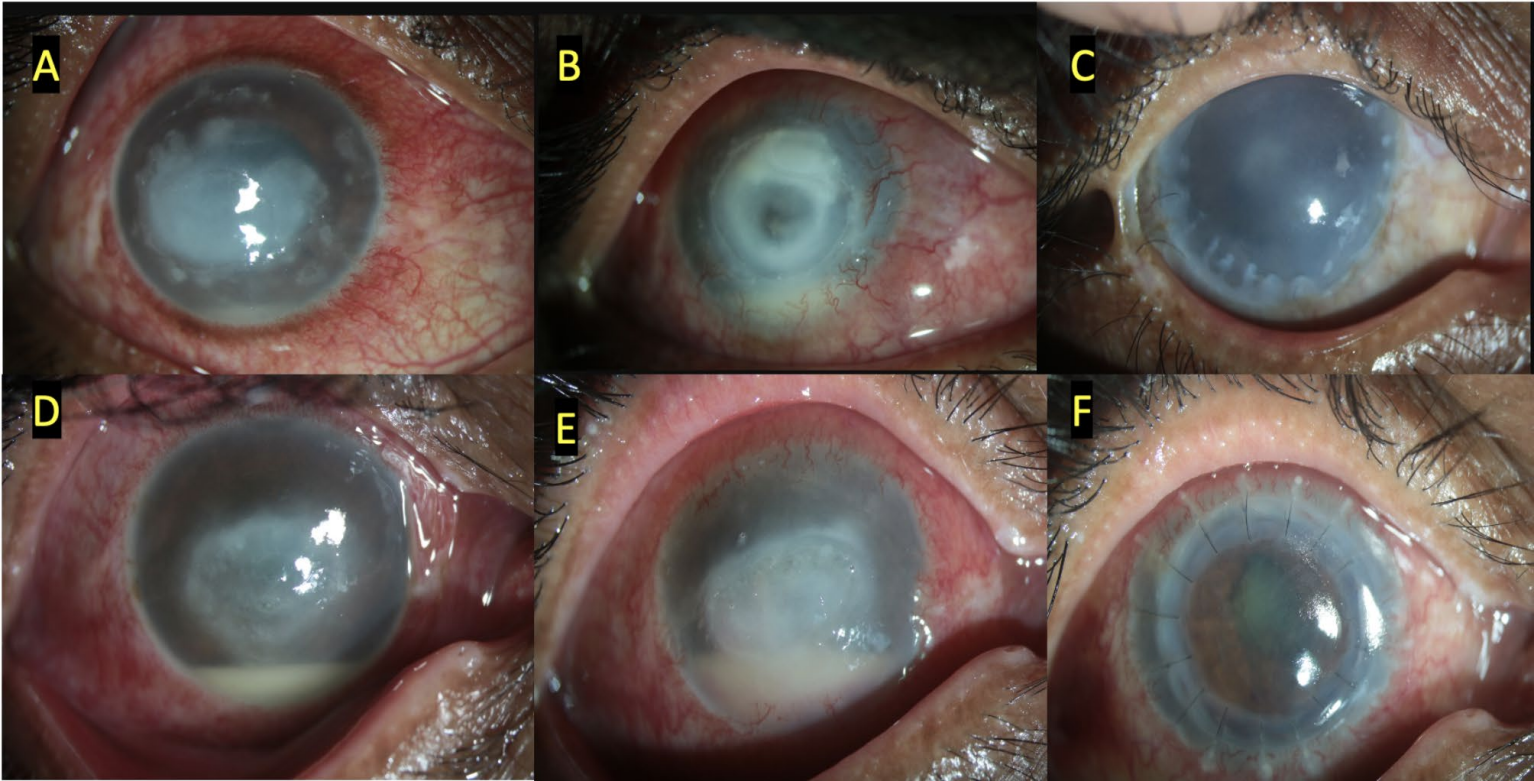
Shows the outcomes of two patients who were managed with topical 0.02% w/v of Polyhexamethylene biguanide and 0.02%w/v Chlorhexidine along with adjuvant PDAT - RB. At presentation (**A** and **D**), At the time of worsening before undergoing PK (**B** and **E**), and after keratoplasty. (**C** and **F**)

**Fig. 3:**
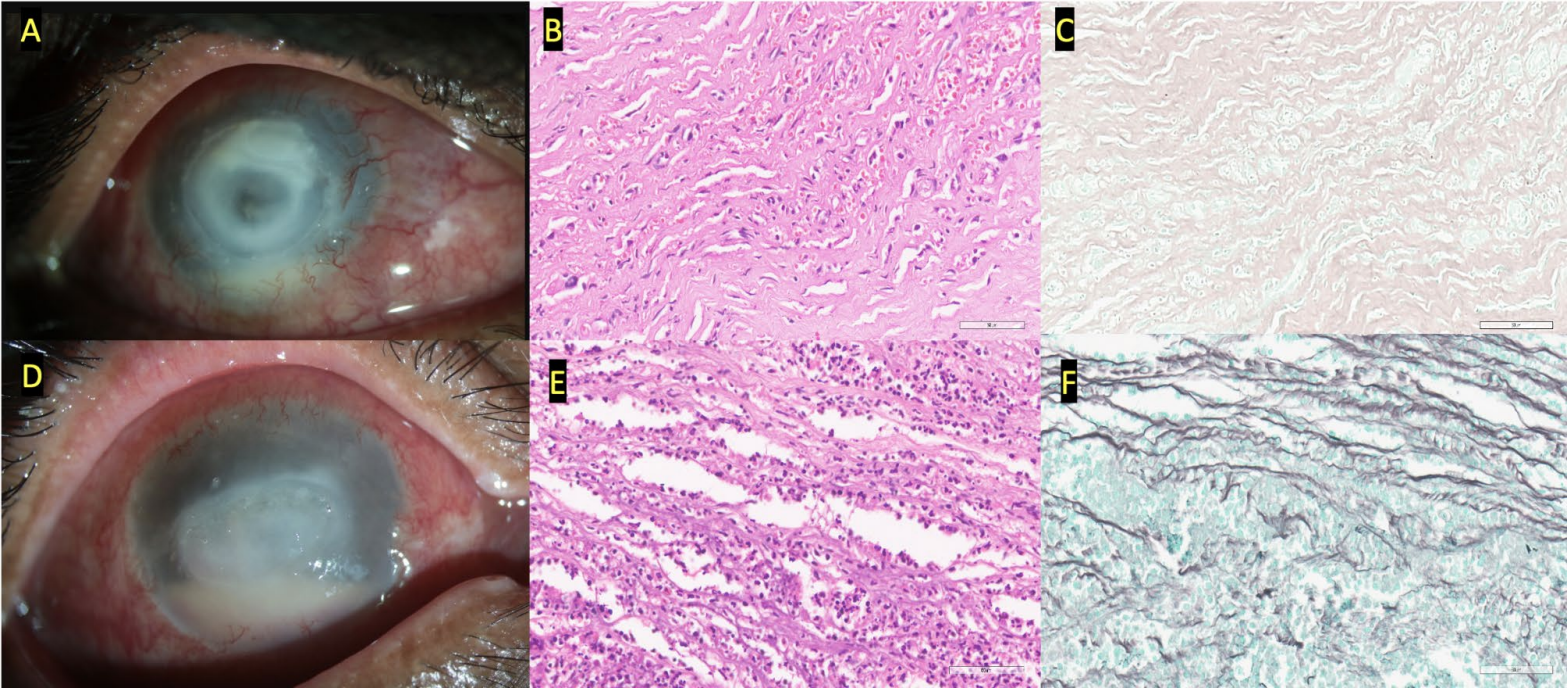
Histopathology of the excised corneal buttons from patients worsened on treatment with adjuvant PDAT-RB, Photomicrographs show – **A**) clinical picture, **B**) moderate stromal infiltrates composed of neutrophils, lymphocytes and plasma cells along with red blood cells (Hematoxylin and Eosin (H&E; 40x original magnification) **C**) GMS stain did not reveal any cysts (GMS; 40x original magnification), **D**) Clinical picture **E**) stroma shows dense mixed inflammatory infiltrates along with exudative debris (H&E; 40x original magnification) **F**) GMS stain does not highlight cysts in the posterior stroma (GMS; 40x original magnification)

On microbiological examination, double-walled cysts of *Acanthamoeba* were observed in 13/14 (92.8%) under direct smear examination, while the growth of *Acanthamoeba* was seen in 10/14 (71.4%) patients in *E.coli* enriched Non-Nutrient Agar media. In none of the cases mixed infections could be found. The median duration between the presentation and 1st session of PDAT was 5 (2.5–10 days). There were a total of 2 sessions performed 1 week apart. Out of 14, in 12 (85.7%) patients, the infection was resolved (Fig. 1) clinically with an average duration of 110 (67.5–150) days. Median LogMAR final visual acuity improved to 1.60 ± 1.3. topical corticosteroids (Prednisolone acetate 1%) were added in 8/14(57.1%) patients. There was no difference in the outcome in terms of resolution with or without steroids. While two patients (Fig. 2) worsened and needed keratoplasty). Histopathology of these corneal buttons showed neutrophilic infiltration but no double-walled cysts (Fig. 3). One of them has the growth of *Acanthamoeba* from the dissected button. The median total follow-up of patients of the PDAT group was 210 (150–370) days. These grafts failed on follow-up and needed Descemet’s stripping endothelial keratoplasty and cataract surgery later.

## Discussion

In the present study, we have reported the outcome of *Acanthamoeba* keratitis with PDAT-RB and the continuation of medical treatment. This study was based on the previously published effect of PDAT-RB in the rabbit model of *Acanthamoeba* keratitis by Atalay et al. [20] and clinical cases published by Naranjo et al. [18]. The uniqueness of our trial is that in all of the 14 patients from our study, PDAT was applied immediately after the confirmation of diagnosis in contrast to previously published series [18], where it was performed in very advanced stage of infection as a measure to avoid TPK. There is a significant improvement in visual acuity, probably explained by faster clinical response and less scarring [21, 22].

The current study also highlights the procedure’s safety in the early stage of infection and its possible adjuvant role in the management of *Acanthamoeba* keratitis. Our study is the first to show the outcome of PDAT -RB in early cases of *Acanthamoeba* keratitis. It can be helpful for further guidance and planning of future prospective studies with a larger sample size. Although worsening of infection was found in 2 (14.2%) cases from our series, there are still good clinical responses noted in 12 cases which is better than the previously published series.

As mentioned in the literature [23] before, corticosteroids can have a detrimental effect on prognosis; we could not find such an impact in our cases in our groups despite having differences. This could be a shorter duration (< 2 weeks) of steroids as compared to the literature. In our series, steroids were administered based on the previously defined criteria, such as in cases pre-treated with AAK for at least 2–3 weeks, in the presence of deep stromal vessels, and/ or associated with severe pain. We have done confocal microscopy of all patients to confirm the diagnosis, and in many of the patients, the scan was repeated on follow-up to observe the effect of PDAT on them. We followed the disappearance of cysts in cases successfully managed with clinical resolution and the presence or increase of cysts in cases with worsening infection [24]. Although our study lacks in vitro data, similar data has been reported by Chen et al. [25] with PDAT on trophozoites and cysts of *Acanthamoeba*, where they found an inhibitory effect of PDAT on the cysts and trophozoites of Acanthamoeba. For observing the additional advantage over medical treatment alone, we have analyzed outcomes from cases (*N* = 27) treated between 2018 and 2019 (unpublished data) with similar clinical profiles, all managed with a combination of PHMB and chlorhexidine alone. Our observations suggest that the addition of adjuvant PDAT provided additional benefits, including a reduced need for urgent therapeutic corneal transplantation (2/14 [7%] vs. 3/27 [11.1%]), faster resolution of infection (median 110 days [67.5–150] vs. 120 days [90–172.5]), and improved final visual acuity. Moreover, the requirement for optical keratoplasty was significantly lower in the PDAT group (0 vs. 6 in the topical treatment-only group). Although there seems to be additional advantage over the medical therapy, there is a scope of prospective randomized study to compare the outcome with adjuvant PDAT + RB with larger sample size to confirm these findings.

**Table 1:** Shows the baseline table 1 shows the baseline information of the patients, with microbiologically confirmed *Acanthamoeba* keratitis, who underwent adjuvant PDAT + RB along with combination of topical PHMB and chlorhexidine, including details of their treatment and outcome

S/*N*	Age (years)	Gender	Treatment at presentation	Duration of onset(Days)	Size of infiltrate (VXH)(mm)	OCT depth(µ)	Duration of PDAT and first Diagnosis(days)	Steroids added after PDAT(y-1, *N*-2)	Steroids added after how many days	Final Outcome	Vision at the last visit
1	44	Female	Natamycin, Moxifloxacin	20	4 × 5	170	9	N	NA	Resolved	20/30
2	28	Male	Moxifloxacin, Prednisolone	30	5 × 4.5	250	5	N	NA	Resolved	20/25
3	38	Male	MoxifloxacinPrednisolone	60	8.3 × 7.5	300	15	y	7	Resolved	20/320
4	72	Male	Natamycin	25	8 × 8	250	11	y	1	Resolved	CFCF
5	40	Male	Moxifloxacin	30	3.4 × 6.2	272	13	y	8	Worsened	CFCF
6	15	Female	Voriconazole	21	8 × 8	300	8	y	18	Resolved	20/20
7	63	Male	Ciprofloxacin	10	6 × 4.5	270	2	N	NA	Worsened	CFCF
8	41	Female	Prednisolone	20	5 × 6.5	300	5	N	NA	Resolved	20/50
9	59	Male	Ciprofloxacin	31	6.5 × 4.6	350	5	N	NA	Resolved	CFCF
10	50	Male	Natamycin, Moxifloxacin	30	4.9 × 5.4	204	1	N	NA	Resolved	CFCF
11	63	Female	Natamycin, Moxifloxacin	30	6 × 6	300	15	y	120	Resolved	CFCF
12	54	Male	Natamycin	30	6 × 6.2	250	1	y	60	Resolved	20/400
13	52	Male	Voriconazole, steroids	20	6 × 6.7	250	0	y	90	Resolved	20/160
14	25	Male	Antivirals, steroids	90	3.3 × 3.5	250	3	y	60	Resolved	20/50

## Data Availability

No datasets were generated or analysed during the current study.
